# Rare variants in genes encoding the cardiac sodium channel and associated compounds and their impact on outcome of catheter ablation of atrial fibrillation

**DOI:** 10.1371/journal.pone.0183690

**Published:** 2017-08-24

**Authors:** Daniela Husser, Laura Ueberham, Gerhard Hindricks, Petra Büttner, Christie Ingram, Peter Weeke, M. Benjamin Shoemaker, Volker Adams, Arash Arya, Philipp Sommer, Dawood Darbar, Dan M. Roden, Andreas Bollmann

**Affiliations:** 1 Department of Electrophysiology, University of Leipzig, Heart Center, Germany; 2 Department of Medicine, Vanderbilt University School of Medicine, Nashville, TN, United States of America; 3 Department of Cardiology, Copenhagen University Hospital Gentofte, Hellerup, DK; 4 Division of Cardiology, University of Illinois, Chicago, IL, United States of America; Pennsylvania State University, UNITED STATES

## Abstract

**Aim:**

Rare variants of genes encoding the cardiac sodium channel and associated compounds have been linked with atrial fibrillation (AF). Nevertheless, current expert consensus does not support genetic testing in AF, which is in part based on the fact that “there is no therapeutic impact derived from AF genetic test results”. However, there are no studies available supporting this recommendation. Consequently, this study analyzed the impact of rare variants affecting the cardiac sodium channel on rhythm outcome of AF catheter ablation.

**Methods and results:**

In 137 consecutive patients with lone AF enrolled in the Leipzig Heart Center AF ablation registry, screening for mutations in *SCN5A*, *SCN1B – 4B*, *CAV3*, *GPD1L*, *SNTA1* and *MOG1* was performed. We identified 3 rare non-synonymous variants in *SCN5A*, 5 in *SCN1B*, 1 in *SCN4B*, 1 in *CAV3*, 6 in *GPD1L*, 3 in *SNTA1* and 3 in *MOG1* (16%). Variant carriers were otherwise comparable with non-variant carriers. Analysis of AF recurrence rates after radiofrequency AF catheter ablation by serial 7-day Holter ECG monitoring between 3 and 12 months revealed no difference between groups, i.e. 45% in variant carriers vs. 49% in non-variant carriers.

**Conclusions:**

Rare variants in genes encoding the cardiac sodium channel and associated compounds are frequently found in lone AF but were not found to impact the outcome of AF catheter ablation. This finding supports current recommendations not to screen for rare variants for the ablation outcome in AF. Nevertheless, it may at least be helpful for understanding AF mechanisms and larger studies are needed to further explore the possible association between genotype and response to AF therapies.

## Introduction

Rare variants of genes encoding the cardiac sodium channel have been linked with atrial fibrillation (AF). For instance, mutations in the *SCN5A* gene that encodes the alpha-subunit of the sodium current (Nav1.5) have been found in 9% of an unselected AF population and in 6% of patients with lone AF.[1] Mutations of the *SCN1B –SCN4B* genes that encode the modifying beta-subunits of the Navß1-ß4 have been detected in an additional 2%.[2, 3] Other genes play an important role for the generation of a healthy Nav1.5 current by coding for accessory channel anchoring or trafficking proteins. Mutations in four of these genes, i.e. *GPD1L*, *MOG1*, *SNTA1*, *CAV3* have been linked with AF [4] and long QT syndrome (LQTS),[5, 6] Brugada syndrome (BrS) [7] or sudden infant death syndrome (SIDS) [8].

However, current expert consensus does not support genetic testing in AF. This recommendation is in part based on the fact that “there is no prognostic or therapeutic impact derived from AF genetic test result”.[9] Since there are no studies available supporting this recommendation we sought to investigate the frequency and potential impact of rare sodium channel or associated compound variants on rhythm outcome of catheter ablation of AF.

## Methods

### Study population

The study included 137 consecutive patients of Caucasian ancestry with drug refractory paroxysmal or persistent lone AF enrolled in the Leipzig Heart Center AF Ablation and Genetics Registry, who underwent AF radiofrequency ablation. The patient characteristics are summarized in Table 1 and S1 Table.

**Table 1:** Rare variants in AF.

Gene	n	Locus of mutation	NCBI Reference Sequence
*SCN5A*	1 1 1	SCN5A/Exon2/c.73G>A/p.E25K SCN5A/Exon9/c.1036G>A/p.E346K SCN5A/Exon17/c.3190G>A/p. E1064K	NM_000335.4
*SCN1B*	3 2	SCN1B/Exon3_ext/c.448+193G>A/p.R214Q	NM_199037.3
		SCN1B/Exon3_ext/c.448+321G>A/p.G257R	
*SCN4B*	1	SCN4B/Exon6/c.632C>G/p.T211R	NM_174934.3
*GPD1L*	4 1 1	GPD1L/Exon4/c.370A>G/p.I124V GPD1L/Exon3/c.267C>A/p.D89E GPD1L/Exon4/c.391C>A/p.L131M	NM_015141.3
*MOG1*	3	MOG1, Exon 1_2/c.181G>T/p.E61X	NM_016492.4
*SNTA1*	1 2	SNTA1/Exon4/c.787G>T/p.A263S SNTA1/Exon3/c.556C>T/p.S189L	NM_003098.2
*CAV3*	1	CAV3/Exon2/c.433G>A/p.V145M	NM_033337.2

Paroxysmal AF was defined as self-terminating episodes of AF within 7 days after onset documented by ECG or an ambulatory ECG monitor. Persistent AF was defined as an AF episode either lasting longer than 7 days or requiring drug or direct current cardioversion for termination.

In all patients, transthoracic and transesophageal echocardiography was performed prior to catheter ablation. Left atrial diameter and left ventricular ejection fraction were determined using standard measurements and a left atrial thrombus was excluded. All class I or III antiarrhythmic medications with the exception of amiodarone were discontinued at least 5 half-lives before the procedure.

The study was approved by the institutional review board (Medical Faculty, Leipzig University) and performed in agreement with the declaration of Helsinki. All patients provided written informed consent for study participation.

### Genotyping

Whole blood was collected for genomic DNA extraction and analysis from all subjects. We directly sequenced the coding regions of high priority candidate ion channel genes *SCN5A* (sodium channel, voltage-gated, type V, alpha subunit), *SCN1B* (sodium channel, voltage-gated, type I, beta subunit), *SCN2B* (sodium channel, voltage-gated, type II, beta subunit), *SCN3B* (sodium channel, voltage-gated, type III, beta subunit), and *SCN4B* (sodium channel, voltage-gated, type IV, beta subunit) as well as non-ion channel candidate genes *SNTA1* (syntrophin, alpha 1), *CAV3* (caveolin 3), *MOG1* (RAN guanine nucleotide release factor) and *GPD1L* (glycerol-3-phosphate dehydrogenase 1-like protein).

Screening for variants was performed by PCR amplification of coding regions and flanking intronic sequences followed by direct sequencing of amplicons on an ABI prism 3730 DNA Sequence Detection System. All variants were validated by re-sequencing an independent PCR-generated amplicon from the subject.

### Variant selection and functional analysis

All non-synonymous variants (missense, nonsense, frame-shift, and splice-site) were screened against dbSNP build 137, National Heart, Lung, Blood Institute (NHLBI) Exome Sequencing Project (ESP) and the Exome Aggregation Consortium (ExAC, http://exac.broadinstitute.org). Variants were defined as being rare if a minor allele frequency < 1% was reported in all of the interrogated databases and variants absent from these were defined as being novel. Single base conservation scores and predicted functional effects were evaluated by PhastCons, Genomic Evolutionary Rate Profiling (GERP), Grantham scores, and PolyPhen2 using the SeattleSeq Genomic Variation Server and are presented in Table 2 and S2 Table.

**Table 2:** Patient characteristics.

	Total	Variant carriers (N = 22)	Variant non-carriers (N = 115)	P-value
Age, yrs	51±11	52±14	51±11	0.595
Male (%)	93	96	93	0.676
Persistent AF (%)	34	41	32	0.427
AF history, months	73±66	74±74	72±65	0.868
BB^†^ / CCB^‡^ use (%)	80	86	78	0.388
Digitalis use (%)	10	18	9	0.241
AAD^§^ use (%)	43	45	43	0.805
LAD^¶^, mm	42±6	43±5	42±6	0.604
LVEF^#^ (%)	61±11	63±11	61±11	0.455

^†^BB—beta blockers

^‡^CCB—calcium channel blockers

^§^AAD—antiarrthythmic drugs

^¶^LAD—left atrial diameter

^#^LVEF—left ventricular ejection fraction.

### Catheter ablation

Left atrial catheter ablation was performed using a previously described approach.[10] In brief, patients were studied under deep propofol sedation with continuous invasive monitoring of arterial blood pressure and oxygen saturation. Non-fluoroscopic 3D catheter orientation, CT image integration, and tagging of the ablation sites were performed using Ensite NavX, Ensite Velocity (St. Jude Medical, St. Paul, MN, USA) or CARTO 3 (Biosense Webster, Diamond Bar, CA, USA). Trans-septal access and catheter navigation were performed with a steerable sheath (Agilis, St. Jude Medical, St. Paul, MN, USA). Patients presenting with AF at the beginning of the procedure were electrically cardioverted and ablation was performed during sinus rhythm (i.e. AF termination with ablation was not attempted). In all patients circumferential left atrial ablation lines were placed around the antrum of the ipsilateral pulmonary veins (irrigated tip catheter, pre-selected tip temperature of 48°C, and maximum power of 30–50 W). In patients with persistent AF, additional linear lesions were added at the left atrial roof, the basal posterior wall and the left atrial isthmus. Ablation of complex fractionated electrograms was not performed.

After circumferential line placement, voltage and pace mapping along the ablation line were used to identify and close gaps. The isolation of all pulmonary veins with bidirectional block was verified with a multipolar circular mapping catheter and was defined as the procedural endpoint.

### Follow-up

After ablation, class I and III antiarrhythmic drugs were not reinitiated. Oral anticoagulation was prescribed for 6 months, and proton pump inhibitors were added for 4 weeks. All patients were followed in the outpatient clinic for 12 months after the ablation. During this follow-up period, 7-day Holter ECG recordings were performed 3, 6 and 12 months after the ablation. Additional ECGs and Holter ECG recordings were obtained when patients’ symptoms were suggestive of AF. AF recurrence was defined as a documented AF episode lasting longer than 30 seconds between 3 and 12 months after the ablation (thus, including a 3-month “blanking period”). All patients with sustained early recurring AF underwent direct cardioversion. Additional drug administration was left to the discretion of the treating physician.

### Statistical analysis

Continuous variables are presented as mean values ± one standard deviation and categorical variables as frequencies. Comparison of continuous variables was performed using the unpaired Student’s t-test and of categorical variables using the Pearson chi-square test. A two-sided p-value < .05 was considered as statistically significant.

Considering a case–control ratio of 1:5 and assuming large variant effects, a difference of 57% versus 25% AF recurrence rates could have been detected with power of 80%.

## Results

### Sodium channel and associated compound rare variants in AF

In 22 patients (16%), we identified 3 rare non-synonymous variants in *SCN5A*, 2 in *SCN1B*, 1 in *SCN4B*, 1 in *CAV3*, 3 in *GPD1L*, 2 in *SNTA1* and 1 in *MOG1* (Table 1). Variant details and *in-silico* prediction is summarized in S2 Table.

There were no significant differences in clinical characteristics between variant carriers and non-variant carriers (Table 2). The individual clinical and ECG characteristics of the variant carriers are summarized in S1 Table. None of the patients exhibited a typical BrS pattern on 12-lead ECG or showed a prolonged QT interval duration suggestive of LQTS. Patients’ history and family history was not suspicious with respect to syncope, aborted sudden cardiac death or SIDS. Three patients each had a brother with AF who either had died or did not consent for genetic testing.

#### Outcome of AF catheter ablation

Complete pulmonary vein isolation as a procedural end point was achieved in all cases. Eleven patients did not have a complete follow-up and were therefore excluded from the analysis. AF recurrence within the first week (ERAF) and between 3 and 12 months (LRAF) were observed in 49% and 48%, respectively.

**Fig 1:**
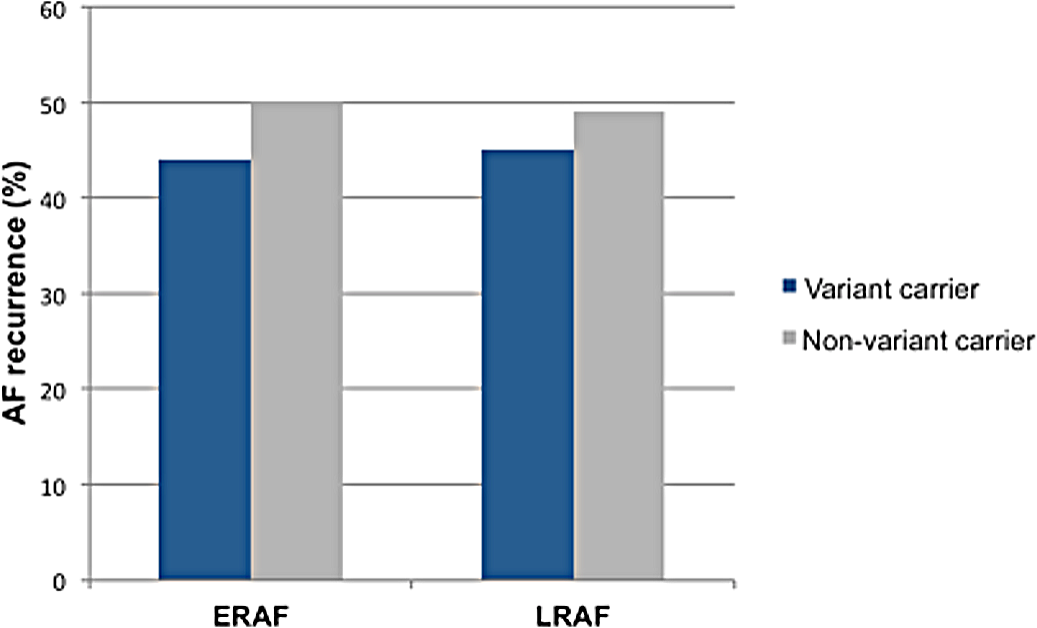
AF recurrence stratified by variant carrier status.

Patients with AF recurrence are compared to patients without AF recurrence in Table 3. There were no differences in clinical characteristics between patients with and without ERAF and LRAF. Patients’ variant carrier status had also no impact on the recurrence of AF (Fig 1). In patients with LRAF, reablation rates were comparable between both groups (78% in variant carriers versus 68% in variant non-carriers, p = .709). All of those patients had reconnected pulmonary veins.

**Table 3:** Comparison of patients with and without early (ERAF) and late recurring AF (LRAF).

	ERAF+ n = 62	ERAF- n = 65	P value	LRAF+ n = 62	LRAF- n = 67	P value
Male %	97	94	0.680	94	94	>0.999
Persistent AF %	32	28	0.613	34	30	0.624
AF history (months)	74 ± 63	77 ± 72	0.958	82± 73	66 ± 60	0.251
Age (years)	52 ± 11	50 ± 12	0.380	53 ± 11	50 ± 11	0.131
BB^†^/CCB^‡^ use %	86	78	0.285	86	78	0.251
Digitalis use %	11	9	0.724	13	9	0.471
Previous AAD^§^ use %	44	45	0.842	48	40	0.355
LAD (mm)	43 ± 6	41 ± 5	0.323	42 ± 6	42 ± 6	0.945
LVEF (%)	61 ± 9	61 ± 13.0	0.480	62 ± 8	61 ± 13	0.933
IVSd^¶^ (mm)	12 ± 2	12 ± 2	0.816	12 ± 2	12 ± 2	0.667
LVED^#^ (mm)	50 ± 6	48 ± 9	0.219	49 ± 9	49 ± 6	0.396
Additional linear lesions (%)	53	44	0.286	56	47	0.323
Ablation time (s)	3177 ± 1433	3158 ± 1117	0.950	3472 ± 1501	3005 ± 1174	0.158
Variant carriers %	13	16	0.662	15	16	0.766

^†^BB—beta blockers

^‡^CCB—calcium channel blockers

^§^AAD—antiarrthythmic drugs

^¶^IVSd—interventricular septal enddiastolic dimension

^#^LVED—left ventricular end diastolic diameter.

## Discussion

### Main findings

To the best of our knowledge, this is the first study to analyze the impact of rare variants in cardiac sodium channel and associated compound genes on outcome after catheter ablation for AF. Analysis of 137 patients with lone AF revealed a prevalence of sodium channel and associated compound gene rare variants of 16%, but no large impact on outcome of ablation.

### Rare variants in genes responsible for the sodium current in AF

The voltage-gated cardiac sodium channel is a multiprotein complex in which accessory proteins interact with the alpha-subunit to regulate its gating, cellular localization, intracellular transport, and degradation. It conducts the inward sodium current (INa) that initiates action potential depolarization and is essential for action potential propagation in the heart but also influences repolarization and refractoriness.[11]

Several genetic variants in *SCN5A*, causing both loss and gain of INa, have been associated with lone AF,[1, 12] thereby expanding the number of phenotypes associated with rare variants in the *SCN5A* gene. Although the fundamental mechanisms of each of these *SCN5A* associated disorders may differ considerably, phenotypic overlap exists, including individuals with both the LQTS and BrS [13] and those with both BrS and conduction system disease.[14] Also, up to 3% of patients with LQTS and 22% with BrS suffer from AF.[15, 16]

Selection of other candidate genes (*SCN1B-4B*, *GPD1L*, *MOG1*, *SNTA1*, *CAV3*) was based on previously described associations between variants in these genes and AF, LQTS or BrS. The encoded accessory proteins play an important role in the generation of healthy cardiac sodium current. The protein encoded by the *GPD1L* gene is found in the cytoplasm, associated with the plasma membrane, where it binds the sodium channel, voltage-gated, type V, alpha subunit. Rare variants in this gene are a cause of BrS type 2 [7] as well as SIDS [8] by decreasing INa through a reduction in *SCN5A* cell surface expression.[7]

The *MOG1* gene plays an important role in sodium channel trafficking by increasing plasma membrane expression of Na(v)1.5 and sodium current (I(Na)) density. A disturbed function due to rare variants and resulting loss-of/reduced sodium current has been shown to cause a BrS type 11, sick-sinus syndrome and lone AF.[4, 17, 18]

The N-terminal domain of the syntrophin protein encoded by the *SNTA1* gene interacts with the C-terminus of the pore-forming alpha subunit of the cardiac sodium channel Nav1.5. This gene is a susceptibility locus for LQTS type 12 and SIDS due to an increase in late sodium current.[19, 20]

Caveolin-3, encoded by *CAV3* is identified as an important negative regulator for cardiac late INa via nNOS dependent direct S-nitrosylation of *SCN5A*, however *CAV3* mutations increase late INa and cause LQTS type 9.[21, 22]

In our cohort, 16% patients were carriers of rare variants. However, aside from lone AF they did not express any particular ECG phenotype, i.e. PR prolongation, LQTS or BrS patterns. In order to predict functionality we reported the results of recognized *in-silico* tools. However, the accuracy of these tools is limited, especially for the sodium channel.[23] There were contradictory results between tools and some variants that had previously been reported in association with BrS [7] and SIDS [8] were reported as “benign”. In that context, it also needs to be pointed out that a comprehensive mutational analysis of 829 unrelated subjects estimated that about 5% of healthy individuals harbor a rare non-synonymous variant in *SCN5A*.[24] Thus, it remains elusive whether or not those rare variants were disease causing.

### Associations between genotype and AF ablation outcomes

Over the last 5 years, several studies have analyzed possible associations between common genetic variants and AF ablation outcomes. Our group was the first to report an association between common AF-susceptibility alleles on chromosome 4q25 and rhythm outcomes of catheter ablation.[25] Meanwhile, this finding has been replicated by another single-center study [26] and very recently by a meta-analysis including 991 patients from 3 centers.[27]

Several other, smaller studies have used a candidate gene approach focusing on different AF pathophysiological pathways. So far, polymorphisms in the soluble epoxide hydrolase gene (*EPHX2*),[28] the heme oxygenase-1 gene (*HO-1*),[29] the angiotensin-converting enzyme gene (*ACE*),[30] the interleukin-6 receptor gene (*IL6R*),[31] and the angiotensinogen gene (*AGT*) [32] have been found to associate with recurring atrial arrhythmias but replication studies are lacking.

All these studies point to the potential role of genotypes for the individual stratification of therapeutic interventions in AF patients with the goal of improving rhythm outcome and increasing patient safety for instance through better patient selection, tailored ablation and post ablation management.

However, unselected screening for rare variants was not helpful for this purpose in our cohort consequently supporting current recommendations [9] and clinical practice. Ablation outcomes in our cohort that represents the largest study with known rare variant carrier status were in agreement with two previous case series in 9 and 5 BrS patients in whom mutations were, however, not reported.[33, 34]

### Limitations

Although we included patients with lone AF, left atrial diameter was somewhat enlarged with low prevalence of diastolic dysfunction with no differences among both groups. Overall we found no large impact of rare variants on outcome of de-novo catheter ablation. Moreover, although reablation rates were comparable with all patients showing reconnected pulmonary veins, the sample size of this subgroup is rather small, therefore, no meaningful comparison of impact on subsequent outcome or AF mechanisms can be made. However, this does not rule out that private mutations may play a role in the outcome of therapeutic interventions for AF. While rare variants are more likely to be associated with large effect sizes, we do acknowledge that functional studies or co-segregation data are required to support disease-causality. Importantly, co-segregation could not be assessed (e.g. 3 brothers were not screened). Moreover, additional functional analysis on any of the detected rare non-synonymous mutations that have not been described previously was not performed. Hence, we are unable to determine whether the identified variants are pathogenic or benign or have smaller effects that could not be detected with this sample size. Although we did not include a non-AF sample, the majority of variants are very rare in reference samples. However, there is no phenotype data available and consequently, controls may in fact contain AF cases or other heart disease.

While we re-sequenced several high priority candidate AF genes, we did not perform whole exome/next generation sequencing. Therefore, we cannot exclude the possibility of our findings being influenced by variants residing in genes that were not screened. It is also possible that by evaluating coding regions only we may have missed potentially important variants in non-coding regions. Lastly, by focusing on rare variants only, we are unable to assess the effects of common variants including combinations of variants, with small or intermediate effects. Furthermore it should be noted that this study applies only to males, since they represent >90% of the study population.

## Conclusions

Rare variants of genes encoding the cardiac sodium channel and associated compounds are frequently found in lone AF but seem not to impact on outcome of AF catheter ablation. This finding is in agreement with current recommendations not to screen for rare variants for the ablation outcome in AF. Nevertheless, it may at least be helpful for understanding AF mechanisms and larger studies are needed to further explore the possible association between genotype and response to AF therapies.

## Supporting information

S1 Table Individual clinical (top), echo- and electrocardiographic (bottom) characteristics of variant carriers. (DOCX)

S2 Table Prevalence of rare variants in other cohorts and functional predictions. (DOCX)

## Abbreviations

AF: atrial fibrillation
LQTS: long QT syndrome
BrS: Brugada syndrome
SIDS: sudden infant death syndrome
*SCN5A*: sodium channel, voltage-gated, type V, alpha subunit
*SCN1B*: sodium channel, voltage-gated, type I, beta subunit
*SCN2B*: sodium channel, voltage-gated, type II, beta subunit
*SCN3B*: sodium channel, voltage-gated, type III, beta subunit
*SCN4B*: sodium channel, voltage-gated, type IV, beta subunit
*SNTA1*: syntrophin, alpha 1
*CAV3*: caveolin 3
*MOG1*: RAN guanine nucleotide release factor
*GPD1L*: glycerol-3-phosphate dehydrogenase 1-like protein
ERAF: early recurrence of atrial fibrillation
LRAF: late recurrence of atrial fibrillation
INa: inward sodium current
*EPHX2*: soluble epoxide hydrolase gene
*HO-1*: heme oxygenase-1 gene
ACE: angiotensin-converting enzyme gene
*IL6R*: interleukin-6 receptor gene
AGT: angiotensinogen gene
BB: beta blockers
CCB: calcium channel blockers
AAD: antiarrthythmic drugs
LAD: left atrial diameter
LVEF: left ventricular ejection fraction
IVSd: interventricular septal enddiastolic dimension
LVED: left ventricular end diastolic diameter
