# Supplementary material for: Rare variants in genes encoding the cardiac sodium channel and associated compounds and their impact on outcome of catheter ablation of atrial fibrillation
Source: PLoS One. 2017 Aug 24;12(8):e0183690. doi: 10.1371/journal.pone.0183690 (PMC5570360; doi:10.1371/journal.pone.0183690)
Supplement: S1 Table — (DOCX) [file pone.0183690.s001.docx]

**S1 Table.** Individual clinical (top), echo- and electrocardiographic (bottom) characteristics of variant carriers.

| **Mutation** | **Gene** | **Gender** | **Age  AF  onset** | **Lone AF** | **History  of  syncope** | **Family history  of AF** | **Family  history  of SCD** | **Family  history  of SIDS** |
| --- | --- | --- | --- | --- | --- | --- | --- | --- |
| Exon2/c.73G>A/p.E25K | SCN5A | m | 42 | y | n | n | n | n |
| Exon9/c.1036G>A/p.E346K | SCN5A | m | 47 | y | n | n | n | n |
| Exon17/c.3190G>A/p.E1064K | SCN5A | m | 55 | y | n | n | n | n |
| Exon3_ext/c.448+193G>A/p.R214Q | SCN1B | m | 43 | y | n | n | n | n |
| Exon3_ext/c.448+193G>A/p.R214Q | SCN1B | m | 56 | y | n | 1 brother | n | n |
| Exon3_ext/c.448+193G>A/p.R214Q | SCN1B | m | 39 | y | n | n | n | n |
| Exon3_ext/c.448+321G>A/p.G257R | SCN1B | m | 53 | y | n | n | n | n |
| Exon3_ext/c.448+321G>A/p.G257R | SCN1B | m | 57 | y | n | n | n | n |
| Exon6/c.632C>G/p.T211R | SCN4B | m | 51 | y | n | n | n | n |
| Exon3/c.267C>A/p.D89E | GPD1L | m | 66 | y | n | n | n | n |
| Exon4/c.370A>G/p.I124V | GPD1L | m | 40 | y | n | n | n | n |
| Exon4/c.370A>G/p.I124V | GPD1L | m | 45 | y | n | n | n | n |
| Exon4/c.370A>G/p.I124V | GPD1L | m | 44 | y | n | n | n | n |
| Exon4/c.370A>G/p.I124V | GPD1L | m | 38 | y | n | n | n | n |
| Exon4/c.391C>A/p.L131M | GPD1L | m | 70 | y | n | n | n | n |
| Exon 1_2/c.181G>T/p.E61X | MOG1 | m | 31 | y | n | 1 brother | n | n |
| Exon 1_2/c.181G>T/p.E61X | MOG1 | m | 12 | y | n | n | n | n |
| Exon 1_2/c.181G>T/p.E61X | MOG1 | f | 70 | y | n | n | n | n |
| Exon4/c.787G>T/p.A263S | SNTA1 | m | 15 | y | n | n | n | n |
| Exon3/c.556C>T/p.S189L | SNTA1 | m | 38 | y | n | n | n | n |
| Exon3/c.556C>T/p.S189L | SNTA1 | m | 61 | y | n | 1 brother | n | n |
| Exon2/c.433G>A/p.V145M | CAV3 | m | 22 | y | n | n | n | n |
| y - yes, n - no, SCD - sudden cardiac death, SIDS - sudden infant death syndrome | | | | | | | | |

| **Mutation** | **Gene** | **LVEF (%)** | **LAD (mm)** | **PQ  interval (ms)** | **QRS duration (ms)** | **Heart rate (bpm)** | **QTc interval (ms)** | **P wave duration (ms)** | **QRS morphology** |
| --- | --- | --- | --- | --- | --- | --- | --- | --- | --- |
| Exon2/c.73G>A/p.E25K | SCN5A | 60 | 37 | 160 | 90 | 62 | 386 | 70 | normal |
| Exon9/c.1036G>A/p.E346K | SCN5A | 63 | 41 | 190 | 80 | 54 | 398 | 85 | normal |
| Exon17/c.3190G>A/p.E1064K | SCN5A | 62 | 41 | 180 | 100 | 60 | 410 | 80 | LBBB |
| Exon3_ext/c.448+193G>A/p.R214Q | SCN1B | 65 | 40 | 150 | 80 | 72 | 416 | 80 | normal |
| Exon3_ext/c.448+193G>A/p.R214Q | SCN1B | 50 | 40 | 140 | 100 | 52 | 391 | 80 | ERP inf, lat |
| Exon3_ext/c.448+193G>A/p.R214Q | SCN1B | 58 | 41 | 140 | 80 | 65 | 360 | 60 | normal |
| Exon3_ext/c.448+321G>A/p.G257R | SCN1B | 65 | 36 | 110 | 100 | 81 | 418 | 60 | ERP inf |
| Exon3_ext/c.448+321G>A/p.G257R | SCN1B | 71 | 46 | 180 | 80 | 70 | 410 | 80 | aRBBB, |
| Exon6/c.632C>G/p.T211R | SCN4B | 76 | 47 | 170 | 80 | 60 | 420 | 80 | aRBBB |
| Exon3/c.267C>A/p.D89E | GPD1L | 60 | 44 | 220 | 100 | 64 | 434 | 80 | ERP inf, lat |
| Exon4/c.370A>G/p.I124V | GPD1L | 60 | 42 | 140 | 80 | 73 | 430 | 70 | normal |
| Exon4/c.370A>G/p.I124V | GPD1L | 65 | 42 | 150 | 90 | 70 | 421 | 60 | aRBBB |
| Exon4/c.370A>G/p.I124V | GPD1L | 65 | 42 | 140 | 100 | 48 | 411 | 80 | normal |
| Exon4/c.370A>G/p.I124V | GPD1L | 60 | 33 | 150 | 100 | 72 | 416 | 80 | normal |
| Exon4/c.391C>A/p.L131M | GPD1L | 60 | 44 | 190 | 100 | 71 | 424 | 80 | normal |
| Exon 1_2/c.181G>T/p.E61X | MOG1 | 64 | 47 | 140 | 80 | 96 | 445 | 80 | normal |
| Exon 1_2/c.181G>T/p.E61X | MOG1 | 60 | 43 | 120 | 80 | 79 | 379 | 40 | normal |
| Exon 1_2/c.181G>T/p.E61X | MOG1 | 50 | 43 | 190 | 90 | 60 | 440 | 70 | ERP inf |
| Exon4/c.787G>T/p.A263S | SNTA1 | 60 | 49 | 160 | 100 | 74 | 411 | 90 | normal |
| Exon3/c.556C>T/p.S189L | SNTA1 | 57 | 50 | 140 | 100 | 78 | 433 | 70 | normal |
| Exon3/c.556C>T/p.S189L | SNTA1 | 50 | 38 | 170 | 80 | 74 | 433 | 90 | ERP inf, lat |
| Exon2/c.433G>A/p.V145M | CAV3 | 70 | 35 | 130 | 80 | 49 | 407 | 70 | ERP inf, lat |

LAD - left atrial diameter, LVEF - left ventricular ejection fraction, iRBBB - incomplete right bundle branch block, LBBB - left bundle branch block, aRBBB - atypical right bundle branch block, ERP - early repolarization pattern, inf - inferior leads, lat - lateral leads
