# Supplementary material for: Rare variants in genes encoding the cardiac sodium channel and associated compounds and their impact on outcome of catheter ablation of atrial fibrillation
Source: PLoS One. 2017 Aug 24;12(8):e0183690. doi: 10.1371/journal.pone.0183690 (PMC5570360; doi:10.1371/journal.pone.0183690)
Supplement: S2 Table — (DOCX) [file pone.0183690.s002.docx]

**S2 Table.** Prevalence of rare variants in other cohorts and functional predictions.

| Chr | Pos | Ref | Alt | Gene/Exon/cDNA/ProteinPosition | accession | function | rsID | PolyPhen2 | Grant- ham  Score | Phast Cons | GERP | ESP  EA4300  Alleles | ESP EA 4300  MAF | ExAC  MAF |
| --- | --- | --- | --- | --- | --- | --- | --- | --- | --- | --- | --- | --- | --- | --- |
| 3 | 8787530 | G | A | CAV3/Exon2/c.433G>A/p.V145M | NM_001234.4 | missense | 142475018 | prob-damaging | 21 | 0.997 | 3.04 | A=1/G=8599 | 0.0001 |  |
| 3 | 32180120 | C | A | GPD1L/Exon3/c.267C>A/p.D89E | NM_015141.3 | missense | NA | benign | 45 | 1 | 3.85 | C=8600 |  |  |
| 3 | 32181723 | A | G | GPD1L/Exon4/c.370A>G/p.I124V | NM_015141.3 | missense | 72552293 | benign | 29 | 0.992 | 3.87 | G=14/A=8586 | 0.0016 | 0.0021 |
| 3 | 32181744 | C | A | GPD1L/Exon4/c.391C>A/p.L131M | NM_015141.3 | missense | NA | benign | 15 | 1 | 5.46 | C=8600 |  | 0.000009 |
| 3 | 38622460 | C | T | SCN5A/Exon17/c.3190G>A/p.E1064K | NM_000335.4 | missense | NA | pos-damaging | 56 | 0.884 | 4.6 | C=8500 |  |  |
| 3 | 38648264 | C | T | SCN5A/Exon9/c.1036G>A/p.E346K | NM_000335.4 | missense | NA | pos-damaging | 56 | 0.164 | -1.21 | T=1/C=8411 | 0.0001 | 0.000008 |
| 3 | 38674726 | C | T | SCN5A/Exon2/c.73G>A/p.E25K | NM_000335.4 | missense | NA | prob-damaging | 56 | 1 | 4.55 | C=8318 | 0.0001 | 0.000008 |
| 11 | 118007797 | G | C | SCN4B/Exon6/c.632C>G/p.T211R | NM_174934.3 | missense | 201454653 | prob-damaging | 71 | 0.929 | 4.46 | A=0/G=8592 |  | 0.0001 |
| 17 | 8192377 | G | T | MOG1/Exon1_2/c.181G>T/p.E61X | NM_001177801.1 | stop-gained | 140704891 | unknown | NA | 0.021 | 4.4 | T=46/G=8550 | 0.0053 | 0.004 |
| 19 | 35524836 | G | A | SCN1B/Exon3_ext/c.448+193G>A/p.R214Q | NM_199037.3 | missense | 66876876 | benign | 43 | 0.005 | 0.783 | A=21/G=4453 | 0.0024 | 0.0033 |
| 19 | 35524964 | G | A | SCN1B/Exon3_ext/c.448+321G>A/p.G257R | NM_199037.3 | missense | 72558028 | benign | 125 | 0 | -2.01 | A=14/G=4372 | 0.0016 | 0.0017 |
| 20 | 32000503 | C | A | SNTA1/Exon4/c.787G>T/p.A263S | NM_003098.2 | missense | 150576530 | benign | 99 | 0.996 | 4.98 | A=1/C=8599 | 0.0001 | 0.0003 |
| 20 | 32005660 | G | A | SNTA1/Exon3/c.566C>T/p.S189L | NM_003098.2 | missense | 144860423 | prob-damaging | 145 | 1 | 5.71 | A=3/G=8597 | 0.0003 | 0.0002 |

ESP EA4300 - exome sequencing project of 4300 individuals of European American ancestry
